# NG2/*CSPG4*, CD146/*MCAM* and VAP1/*AOC3* are regulated by myocardin-related transcription factors in smooth muscle cells

**DOI:** 10.1038/s41598-021-85335-x

**Published:** 2021-03-16

**Authors:** Catarina Rippe, Björn Morén, Li Liu, Karin G. Stenkula, Johan Mustaniemi, Malin Wennström, Karl Swärd

**Affiliations:** 1 Department of Experimental Medical Science, BMC D12, Lund University, 22184 Lund, Sweden; 2 Department of Clinical Sciences, Malmö, Lund University, 221 84 Lund, Sweden; 3 Department of Urology, Qingyuan People’s Hospital, The Sixth Affiliated Hospital of Guangzhou Medical University, Qingyuan, China

**Keywords:** Biochemistry, Cell biology, Computational biology and bioinformatics, Molecular biology, Physiology, Biomarkers

## Abstract

The present work addressed the hypothesis that NG2/*CSPG4*, CD146/*MCAM*, and VAP1/*AOC3* are target genes of myocardin-related transcription factors (MRTFs: myocardin/*MYOCD*, MRTF-A/*MKL1*, MRTF-B/*MKL2*) and serum response factor (*SRF*). Using a bioinformatics approach, we found that *CSPG4*, *MCAM*, and *AOC3* correlate with *MYOCD*, MRTF-A/*MKL1*, and *SRF* across human tissues. No other transcription factor correlated as strongly with these transcripts as *SRF*. Overexpression of MRTFs increased both mRNA and protein levels of *CSPG4*, *MCAM*, and *AOC3* in cultured human smooth muscle cells (SMCs). Imaging confirmed increased staining for CSPG4, MCAM, and AOC3 in MRTF-A/*MKL1*-transduced cells. MRTFs exert their effects through SRF, and the *MCAM* and *AOC3* gene loci contained binding sites for SRF. SRF silencing reduced the transcript levels of these genes, and time-courses of induction paralleled the direct target *ACTA2*. MRTF-A/*MKL1* increased the activity of promoter reporters for *MCAM* and *AOC3*, and transcriptional activation further depended on the chromatin remodeling enzyme KDM3A. *CSPG4*, *MCAM*, and *AOC3* responded to the MRTF-SRF inhibitor CCG-1423, to actin dynamics, and to ternary complex factors. Coincidental detection of these proteins should reflect MRTF-SRF activity, and beyond SMCs, we observed co-expression of CD146/*MCAM*, NG2/*CSPG4*, and VAP1/*AOC3* in pericytes and endothelial cells in the human brain. This work identifies highly responsive vascular target genes of MRTF-SRF signaling that are regulated via a mechanism involving KDM3A.

## Introduction

Myocardin-related transcription factors (MRTFs: *MYOCD*, MRTF-A/*MKL1*, and MRTF-B/*MKL2*) are strong activators of a subset of serum response factor- (SRF) dependent target genes that are relevant for contractility, motility, and adhesion^1–5^. The founding member of this coactivator family is myocardin (official gene symbol *MYOCD*)^3^. MYOCD plays an essential role for cardiac and smooth muscle cell (SMC) development^6,7^ and in adult life^8^. Moreover, MRTF-SRF signaling is critical in endothelial cells (ECs), influencing vascularization and barrier function^9,10^. MYOCD is constitutively active, whereas MRTF-A and MRTF-B shuttle between the nucleus and the cytoplasm depending on the polymerization state of cellular actin; monomeric actin retains MRTFs in an inactive state and actin polymerization promotes nuclear import and target gene activation^2,4,11,12^. Interestingly, MRTFs are mechanosensitive, due in part to their actin-dependence^13,14^. Recent studies have catalogued target genes of MRTFs using transcriptomics^15–19^, and it has become clear that both protein-coding as well as non-coding transcripts, including microRNAs^20–22^ and long non-coding RNAs^19^, are regulated. Currently, comprehensive knowledge of the proteomic impact of MRTFs is incomplete.

Products of classical myocardin target genes, including the myofilament proteins smooth muscle actin (*ACTA2*) and myosin (*MYH11*), are used to identify SMCs in tissue sections by immunological techniques^23^. MYH11, and possibly ACTA2, thus represent cell lineage markers that are characteristic for a specific cell type^23^. Cell lineage markers are useful because their gene regulatory elements can be exploited to create cell-specific knockouts^24^ and for lineage tracing^23,25^. In lineage tracing, promoter or enhancer elements are used to drive DNA recombination events that irreversibly mark cells and their progeny^26,27^. Cell lineage markers are also used in immunohistochemistry to label and enumerate specific cells, but this should be done cautiously when gene regulatory mechanisms are poorly understood^25^.

In recent studies, we leveraged RNA-Sequencing (RNA-Seq) data^28–30^ to define a myocardin/*MYOCD* co-expression module in man. Represented in this group of genes^30^ were those that classify as cell lineage markers, and that have been used to identify and quantify oligodendrocyte progenitors and pericytes (neuron-glial antigen 2: NG2, official gene symbol *CSPG4*)^31,32^, ECs and mesenchymal stem cells (cluster of differentiation 146: CD146, *MCAM*)^33,34^, and vascular cells and myofibroblasts (amine oxidase, copper containing 3 or VAP1, *AOC3*)^35^. The aim of the current study was to test whether *CSPG4*, *MCAM*, and *AOC3* are regulated by MRTFs in human SMCs.

## Results

### NG2/CSPG4 correlates with MRTFs across human tissues

**Figure 1:**
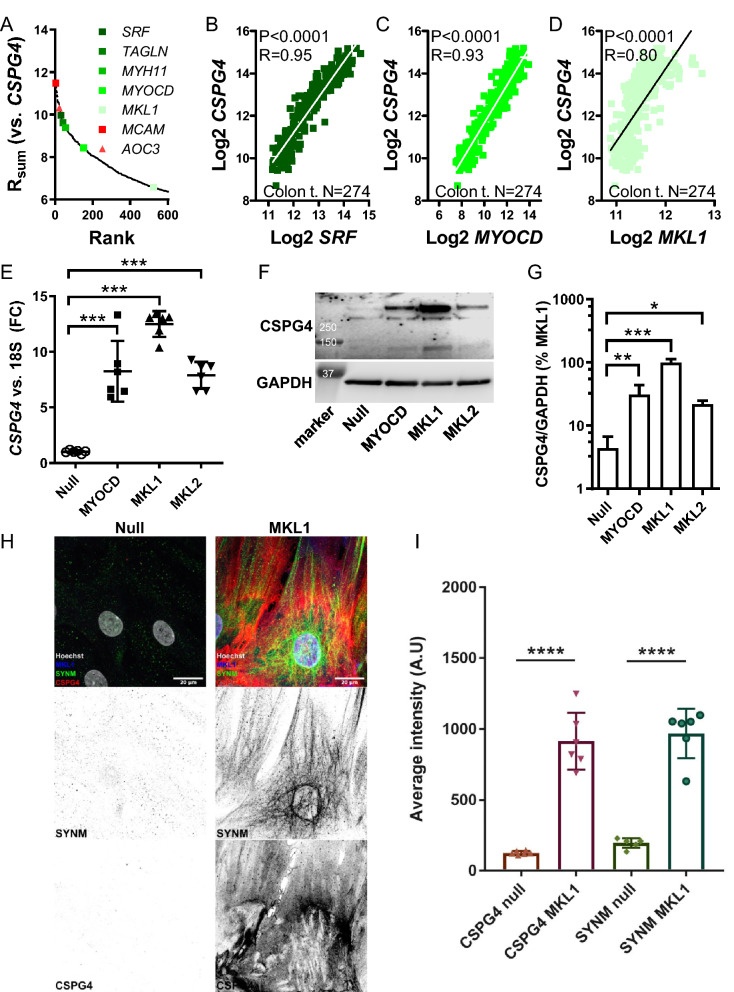
The *CSPG4* gene is activated by Myocardin-Related Transcription Factors (MRTFs). To examine the transcriptional control of NG2/*CSPG4* we correlated the *CSPG4* mRNA versus all other mRNAs (www.GTExPortal.org) and calculated the sum of correlation coefficients (R_sum_) for all transcripts across 20 human tissues. Panel (**A**) shows the positive extreme of the resulting R_sum_ distribution which has a theoretical maximum of 20 (seen only for *CSPG4* itself, not included). Among the 600 (≈1%) most tightly correlating mRNAs we found classical smooth muscle cell (SMC) markers (*TAGLN*, *MYH11*) and transcription factors (*SRF*, *MYOCD*, *MKL1*, all indicated by different green symbols). Two cell lineage markers (*MCAM* and *AOC3*, red symbols) were among the mRNAs in the absolute extreme (top 25). Examples of correlations between *SRF*, *MYOCD* and *MKL1* versus *CSPG4* in the transverse colon (N = 274) are shown in panels (**B**–**D**). P-values and Spearman Rho-coefficients are given in the respective panels. In panel (**E**), adenoviruses were used for overexpression of MRTFs (MYOCD, MRTF-A/*MKL1*, and MRTF-B/*MKL2*) in primary human coronary artery smooth muscle cells in vitro. The *CSPG4* mRNA level was determined by RT-qPCR and compared to that in cells treated with empty virus (ANOVA, followed by Dunnett's Multiple Comparison Test versus Null, N = 6 for all treatments). In this and the following figures showing RT-qPCR data, the relative mRNA level is represented by the official gene symbol in italics. Transcript levels were normalized to 18S as housekeeping gene (Pfaffl equation) and are given as fold change (FC). All statistical comparisons in panel (**E**) of figures 1 through 3 are versus Null as indicated by brackets. Panel (**F**) shows a western blot for NG2/CSPG4 following treatment with control (Null) virus and viruses encoding MRTFs. Membranes were cut horizontally in this, and the following, figures to allow for detection of multiple proteins on the same membrane. Full length blots (as full as possible) are found in Fig. S4. Panel (**G**) shows summarized data for the western blot experiments (N = 3). The bands migrating at ≥ 250 kDa were included in the analysis. In panel (**H**), cells were transduced with tagged MRTF-A/MKL1 (blue), fixed at 96 h, and stained for NG2/CSPG4 (red) and the intermediate filament synemin/SYNM (green), followed by confocal imaging. Red and green labels are shown in black and white below the colored panels for clarity. Summarized data from two independent experiments with three independent replicates each time (N = 6) is shown in panel (**I**). *P < 0.05, **P < 0.01, ***P < 0.001, ****P < 0.0001, all versus Null.

We recently used bioinformatics analyses to find that the cell lineage marker *CSPG4* (a.k.a. neuron-glial antigen 2 or NG2) resides among transcripts that correlate with myocardin/*MYOCD* across human tissues^30^. Here, we extended this analysis using a more recent download of RNA-Seq data (from the GTExPortal.org^28,29^). Doing so we correlated *CSPG4* (NG2) versus all other transcript in a wide selection of human tissues (N = 20 tissues, > 90 individuals per tissue). The sums of Pearson correlation coefficients for all transcripts (N = 56,202) across tissues were calculated and sorted in descending order. The positive extreme (top 600) of the resulting distribution is plotted in Fig. 1A. Several myocardin targets, including *TAGLN* and *MYH11* (green symbols in Fig. 1A), were present in the extreme, as was myocardin itself (*MYOCD*) and its binding partner *SRF*. Also present in the extreme were the myocardin family member MRTF-A/*MKL1* (light green symbol), melanoma cell adhesion molecule (*MCAM*, a.k.a. CD146), and amine oxidase copper containing 3 (*AOC3*, a.k.a. VAP1, red symbols in Fig. 1A). MRTF-B/*MKL2*, and lysine demethylase 3A (*KDM3A*), which are considered further below, had low ranks (rank 10,080 and 10,600). Individual correlations from the extreme were plotted for transverse colon (Fig. 1B–D). The corresponding correlations in arteries were also positive and significant (P < 0.0001, not depicted). Thus, NG2/*CSPG4* correlates with *MYOCD*, *MRTF-A*, and *SRF* across human tissues and in human arteries, and, from a computational point of view, NG2/*CSPG4* is highly co-expressed with *MCAM* and *AOC3* at the transcript level.

### NG2/CSPG4 is induced by adenoviral overexpression of MRTFs

The correlations depicted in Fig. 1B–D may arise because *CSPG4* is a transcriptional target of MRTF-SRF signaling. To test this, we transduced human coronary artery SMCs (hCASMCs) with control virus (Ad-CMV-Null) or with viruses encoding MRTFs (*MYOCD*, MRTF-A/*MKL1*, or MRTF-B/*MKL2*). We then assayed the *CSPG4* mRNA level by reverse transcription quantitative polymerase chain reaction (RT-qPCR). All MRTFs increased the *CSPG4* transcript (8–12-fold, Fig. 1E). In western blots with a specific NG2/CSPG4 antibody, we observed two high molecular weight bands (> 250 kDa) that increased following viral transduction of MRTFs (Fig. 1F and summarized data in G). Imaging of cells by confocal fluorescence microscopy (Fig. 1H) revealed that the NG2/CSPG4 signal (red) increased by about eightfold after MRTF-A/*MKL1* transduction. This increase is comparable to that seen with the positive control synemin/*SYNM*, an intermediate filament protein targeted by all MRTFs^36^ (green staining in Fig. 1H, summarized data in Fig. 1I). Taken together, these findings argue that the cell lineage marker NG2/*CSPG4* is regulated by MRTFs in human SMCs.

### MCAM and AOC3 are also targeted by MRTFs

**Figure 2:**
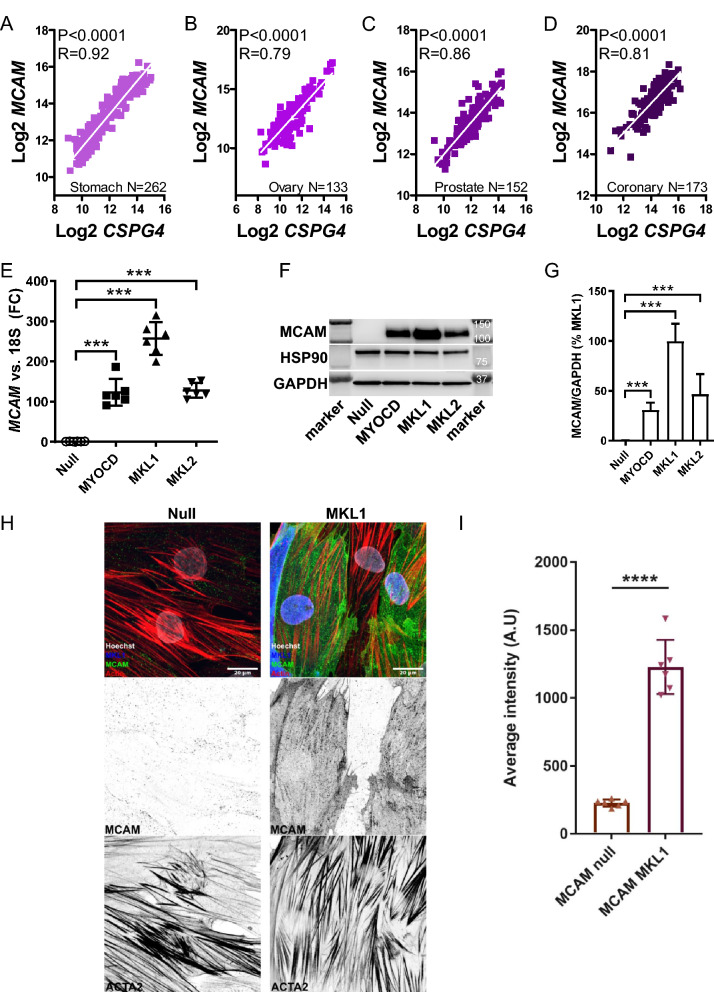
CD146/*MCAM* correlates with *CSPG4* across human tissues and is increased by MRTFs. Panels (**A**–**D**) show examples of correlations between *CSPG4* and *MCAM* in four human tissues (stomach, ovary, prostate, and coronary artery). N-values, P-values, and Spearman Rho-coefficients are given in the graphs. In panel (**E**), MRTFs were overexpressed using adenoviruses and *MCAM* levels were determined by RT-qPCR (N = 6 throughout). Statistical comparisons are versus Null (here and in panel **G**). Panel (**F**) shows a western blot for CD146/*MCAM* and panel (**G**) shows summarized western blot data (N = 6). Panel (**H**) shows confocal imaging of CD146/*MCAM* (green) following overexpression of MRTF-A/*MKL1*. Smooth muscle α-actin (*ACTA2*) is shown in red, and blue represents MRTF-A/*MKL1*. Panel (**I**) shows summarized data on MCAM fluorescence in control cells (Null) and after overexpression of MRTF-A/*MKL1* (N = 6). ***P < 0.001, ****P < 0.0001 versus null.

CD146/*MCAM* was among the top-ranking transcripts in the *CSPG4* co-expression module (c.f. Fig. 1A, red square, and examples in Fig. 2A–D). Therefore, we also addressed if this gene is regulated by MRTFs. The MCAM mRNA (Fig. 2E) and protein levels (Fig. 2F,G) were increased by all MRTFs, with MRTF-A/*MKL1* causing the greatest increase (≈250-fold at the mRNA level). Imaging demonstrated that viral transduction of MRTF-A/*MKL1* also increased MCAM fluorescence (Fig. 2H, green, summarized data in 2I), which was higher than the positive control used in these experiments (smooth muscle α-actin/*ACTA2*, Fig. 2H and data not shown). Therefore, CD146/*MCAM* is also regulated by MRTFs.

**Figure 3:**
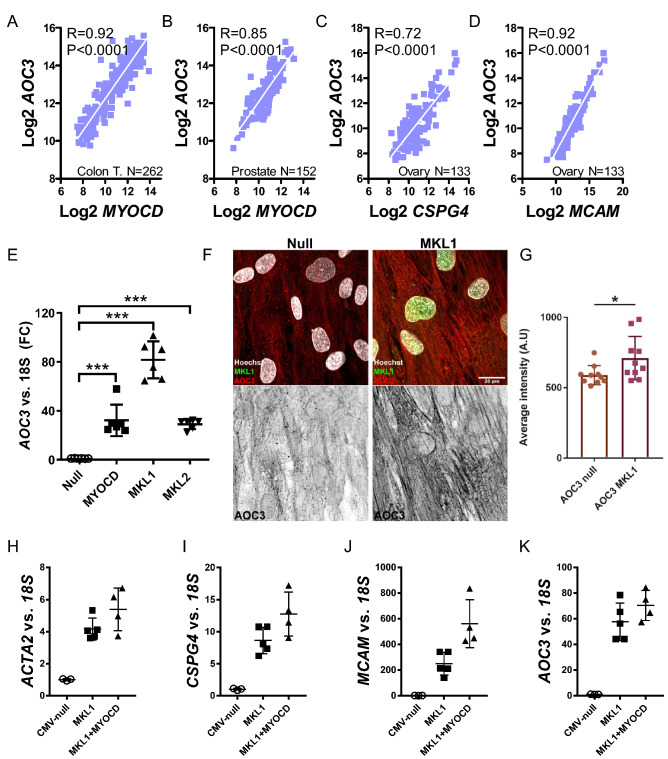
*AOC3* also resides in the *CSPG4* co-expression module and is regulated by MRTFs. Panels (**A**) and (**B**) show correlations between *MYOCD* and *AOC3* in the colon and prostate, respectively. Panels (**C**) and (**D**) show that *AOC3* also correlates with *CSPG4* and *MCAM* (ovary). Panel (**E**) shows mRNA levels for AOC3 following overexpression of MRTFs (all statistical comparisons versus Null). Panel (**F**) shows confocal imaging of AOC3 fluorescence in cells transduced with Null virus and with MRTF-A/MKL1-encoding virus. Panel (**G**) shows summarized data from the experiments in (**F**). Panels (**H**) through (**K**) examine if MYOCD is antagonistic with MRTF-A/*MKL1*. No antagonism was noted for the classical target gene *ACTA2* (**H**), for *CSPG4* (**I**), for *MCAM* (**J**) or for *AOC3* (**K**). *P < 0.05, ***P < 0.001, versus null.

The last transcript in the *CSPG4* co-expression module (in Fig. 1A) that we examined was *AOC3* (a.k.a. VAP1). The *AOC3* mRNA correlated with *MYOCD* (Fig. 3A,B) as well as with *CSPG4* (Fig. 3C) and with *MCAM* (Fig. 3D). Overexpression of MRTFs increased the mRNA level of *AOC3* (30–80-fold, Fig. 3E). Using a non-commercial antibody (TK10-79, kind gift from M. Salmi), and cells rapidly fixed in ice cold acetone, we could also demonstrate a modest increase of AOC3 fluorescence following MRTF-A/MKL1 transduction (Fig. 3F, and summarized data in G). We conclude that NG2/*CSPG4*, CD146/*MCAM*, and VAP1/*AOC3* are regulated by MRTFs in human smooth muscle cells.

So far, our results suggest, but do not prove, that MRTF-A/*MKL1* is a more effective coactivator of *CSPG4*, *MCAM*, and *AOC3* than MYOCD and MRTF-B/*MKL2*. However, MRTF levels may differ after overexpression, and are difficult to compare due to lack of reliable myocardin antibodies^37^. Comparison of the cycle threshold (Ct) values after overexpression suggests that MRTF-B reaches a higher level after overexpression than MRTF-A, which in turn reaches a higher level after overexpression than MYOCD (not shown). MRTF-A is therefore likely a more efficacious transactivator of these genes than MRTF-B. However, the apparently smaller effect of MYOCD could be due to fewer copies of MYOCD mRNA and protein (compared to MRTF-A/*MKL1*). We have previously seen that MRTFs may antagonize each other when transactivation efficacy differs^38^. However, no antagonism could be demonstrated on combined overexpression of MRTF-A/*MKL1* and *MYOCD*, compared to overexpression of MRTF-A/*MKL1* alone (Fig. 3H–K, P > 0.05 for MKL1 vs. MKL1 + MYOCD throughout). Thus, while MRTF-A is likely more effective than MRTF-B, little can be said regarding the relative efficacies of MRTF-A/*MKL1* versus MYOCD.

### Mechanistic studies

**Figure 4:**
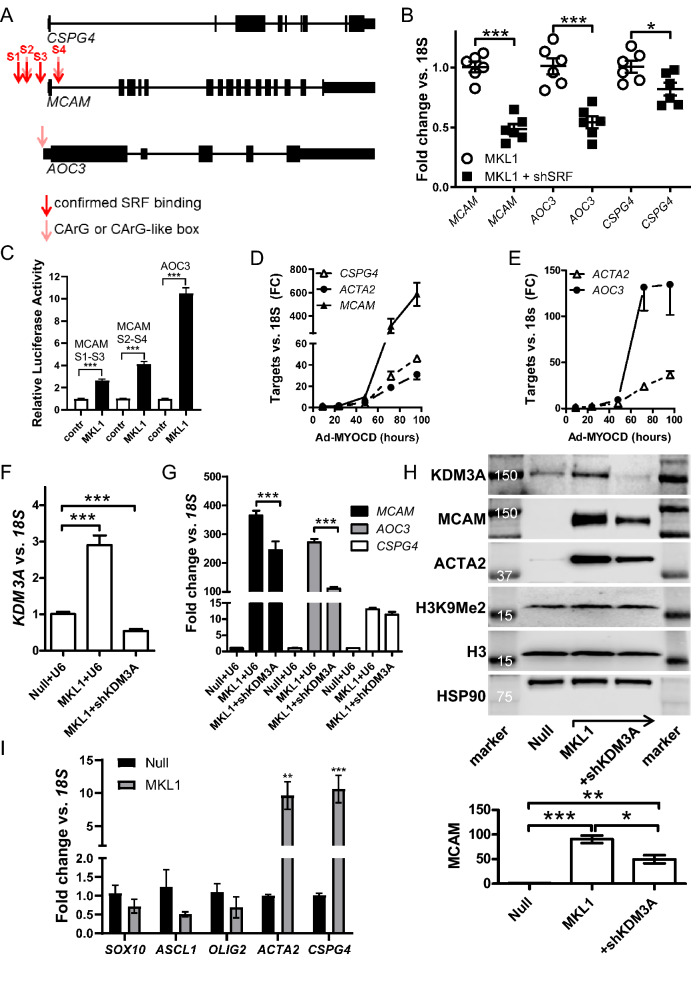
The *MCAM* and *AOC3* gene loci have SRF binding motifs that confer responsiveness to MRTF-SRF signaling. Panel (**A**) shows graphical representations of the *CSPG4*, *MCAM* and *AOC3* gene loci with known (red arrows, ENCODE data accessible via the UCSC genome browser) and predicted (lighter red/pink arrows) binding sites for MRTF-SRF^39^. Gene loci and binding sites are not drawn to scale. Panel (**B**) shows effects of SRF silencing (by 43 ± 4%, P < 0.0001, N = 6), using a short hairpin construct, on the indicated mRNA levels measured by RT-qPCR. Panel (**C**) shows reporter assays for *MCAM* (S1-S3 plasmid and S2-S4 plasmid, see panel **A**) and *AOC3*. We used HEK 293 cells for transfection of the reporter plasmids in (**C**), because these cells were more readily transfected compared to human coronary artery smooth muscle cells used elsewhere. Panels (**D**) and (**E**) show time-courses of mRNA induction following overexpression of myocardin (N = 3 for all time points). *CSPG4*, *MCAM* and *AOC3* were all increased at least as fast as the direct target gene *ACTA2*. Panel (**F**) shows that MRTF-A/MKL1 increases the mRNA level of KDM3A, and that short hairpin silencing of KDM3A (shKDM3A) antagonizes this effect (N = 5–6). Panel (**G**) shows RT-qPCR for *MCAM*, *AOC3*, and *CSPG4* in MRTF-A/MKL1-transduced cells in the absence and presence of shKDM3A (N = 5–6). Panel (**H**) shows western blots for cells transduced with null, MKL1, and MKL1 plus shKDM3A viruses. The bar graph at the bottom shows the quantitative analysis for MCAM (vs. HSP90). Quantitative analysis of the KDM3A protein level similarly showed it to be significantly increased by MRTF-A transduction and reduced by KDM3A silencing (not shown). Panel (**I**) shows the effect of MRTF-A/MKL1 on transcript levels of three transcription factors (*SOX10*, *ASCL1*, and *OLIG2*) that control *CSPG4* expression in brain glial cells. None of these transcription factors were significantly increased, while the positive controls (*ACTA2*, *CSPG4*) were increased in the same samples (N = 6).

Genes targeted by MRTFs have so called CArG-boxes (CC-A/Tx6-GG) to which serum response factor (SRF) binds. We inspected chromatin immunoprecipitation-sequencing (ChIP-Seq) data for SRF (ENCODE) at the three gene loci using the UCSC Genome Browser (screenshots from these analyses are shown in Fig. S1A–C). No SRF binding was noted at the *CSPG4* and *AOC3* loci (Fig. 4A shows diagrammatical representations of the gene loci), but several binding sites were present at the *MCAM* locus (dark red arrows in Fig. 4A middle, called S1-S4). The *AOC3* locus harbored a computationally identified CArG^39^ (Fig. 4A, lighter red arrow). For *MCAM*, two computationally identified CArGs mapped to two of the experimentally determined SRF binding regions (S2 and S4). The presence of CArGs coinciding with binding of SRF in the promoters suggested involvement of SRF. Thus, we examined the role of SRF using short hairpin silencing (shSRF). Silencing of SRF reduced the mRNA levels of *MCAM*, *AOC3*, and *CSPG4* in MRTF-A/MKL1 transduced cells (Fig. 4B). A modest effect was seen on *CSPG4*, probably because knockdown of SRF was incomplete (− 43 ± 4%, P < 0.0001). Limited SRF knockdown is to be expected because it is important for the cell cycle, and SRF knockdown in culture should select for cells where it was less effectively reduced. SRF silencing was not done in the absence of MRTF-A/*MKL1* transduction because *MCAM* and *AOC3* levels were low under basal culture conditions. Overall, these findings support involvement of SRF in MRTF-driven expression of these genes.

We next aimed to verify a regulatory role of the CArGs identified. Because there is no documented SRF binding at the *CSPG4* locus, we focused on *MCAM* and *AOC3*. Two DNA regions, each containing three out of four of the SRF binding sites in *MCAM* (S1–S3 and S2–S4), were used to create promoter reporters. For *AOC3*, we used a commercial reporter plasmid covering ≈1 kb of the proximal promoter and including the computationally identified CArG. We then examined if the respective constructs responded to MRTF-A/*MKL1*. As shown in Fig. 4C, both *MCAM* reporters (S1–S3 and S2–S4) were activated by MRTF-A/*MKL1*, and the *AOC3* reporter was similarly activated (almost tenfold, Fig. 4C). However, reporter activation appeared smaller than effects seen at the mRNA level (compare the 80-fold increase of *AOC3* mRNA with the tenfold increase of promoter activity). This difference suggested either that (1) DNA regions beyond those included in the reporters are involved in regulation, or (2) epigenetic effects requiring intact chromatin are involved, or (3) indirect effects play a role. To address the last possibility, we performed time-course studies for comparison with the direct target gene *ACTA2*^40^. We reasoned that indirect effects should be reflected in a delayed transcript elevation relative to the direct target *ACTA2*, whereas a direct effect should be at least as fast. Interestingly, *CSPG4*, *MCAM* (Fig. 4D) and *AOC3* (Fig. 4E) were increased at least as fast as *ACTA2* following overexpression of myocardin.

We also considered histone acetylation (see H3K27Ac track in Fig. S1A–C), but this was not helpful in terms of identifying additional regulatory mechanisms, and treatment of cells with the deacetylase inhibitor trichostatin A only modestly increased MRTF-A-driven *MCAM* levels, leaving the other transcripts unaffected (Fig. S2).

Histone methylation represents another epigenetic mechanism, and SRF is known to interact with the lysine demethylase KDM1A^41^, while MRTFs (MRTF-A > MYOCD > MRTF-B) interact with KDM3A to activate transcription^42^. We focused on KDM3A because KDM3A was reported in previous work to bind a genomic region flanking the *MCAM* transcription start site (between S3 and S4), and to regulate *MCAM* expression^43^. This raised the possibility that MRTFs exert their effects on *MCAM* in part via KDM3A. In keeping with this possibility, we found that MRTF-A/*MKL1* increased the mRNA level of *KDM3A* (Fig. 4F), and that silencing of KDM3A using a short hairpin construct mitigated MRTF-driven induction of *MCAM* and *AOC3* (Fig. 4G). A similar dependence on KDM3A was seen for classical SMC markers (not shown). MRTF-A/*MKL1* also induced KDM3A at the protein level (Fig. 4H, top membrane), and KDM3A knockdown reduced KDM3A as well as MCAM (Fig. 4H, first and second membranes, plus bar graph at the bottom of Fig. 4H) but left the global level of dimethylated histone H3 (H3K9Me2) unchanged (Fig. 4H). Taken together, these findings suggest that regulation of *MCAM* and *AOC3* by MRTF-A/*MKL1* involves, in part, a local and KDM3A-dependent epigenetic mechanism.

Previous work implicated the transcription factors SOX10, ASCL1, and OLIG2 in regulation of *CSPG4* transcription in the brain^44^. We therefore tested if these transcription factors were increased by MRTF-A/MKL1. Overexpression of MRTF-A/MKL1 had no effect on *SOX10*, *ASCL1*, and *OLIG2*, whereas the positive controls (*ACTA2* and *CSPG4*) increased as expected (Fig. 4I). This argues against an indirect effect mediated by any of these transcription factors.

### CSPG4, MCAM and AOC3 levels change during smooth muscle differentiation, after depolymerization of actin, and following knockout of ternary complex factors

**Figure 5:**
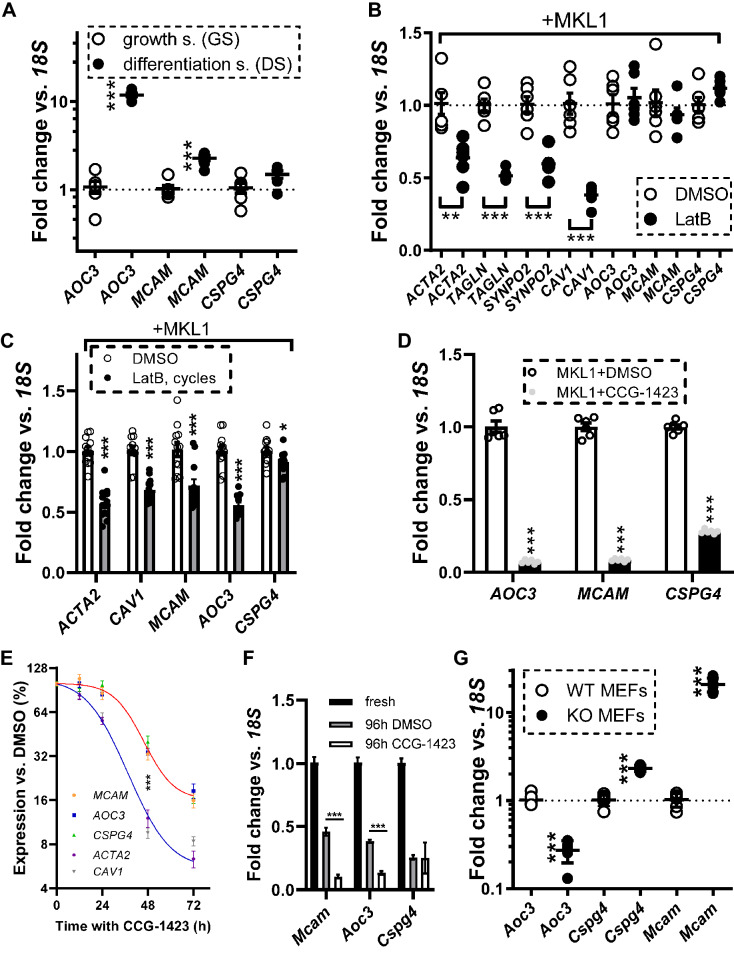
SMC differentiation, actin dynamics, and loss of Ternary Complex Factors (TCFs) all affect *AOC3*, *MCAM,* and *CSPG4* expression. In panel (**A**), human coronary artery smooth muscle cells were incubated with either growth supplement (GS) or differentiation supplement (DS) for 72 h and the mRNA levels of *AOC3*, *MCAM*, and *CSPG4* were determined by RT-qPCR (N = 6). In panels (**B**) and (**C**), the effects of Latrunculin B (LatB), which depolymerizes actin, were tested using two different protocols (**B** 24 h static, and **C** 20 h with drug + 4 h washout in cycles for 96 h, N > 6). Transcript levels were assayed using RT-qPCR. All cells in (**B**) and (**C**) were transduced with MRTF-A/*MKL1*. Panel (**D**) (72 h treatment) and panel (**E**) (time-course) show effects of the MRTF-SRF inhibitor CCG-1423. Panel (**F**) compares transcript levels in freshly isolated mouse caudal arteries with mouse caudal arteries that were organ cultured in the presence of vehicle (DMSO) or CCG-1423 (96 h). In panel (**G**), the levels of *Aoc3*, *Mcam*, and *Cspg4* were determined by RT-qPCR in mouse embryonic fibroblasts (MEFs) from wild type (WT) mice, and in MEFs from mice that lack three Ternary Complex Factors (Elk1, Elk3, and Elk4). *P < 0.05, **P < 0.01, ***P < 0.001, versus the respective controls.

We next examined if incubation of hCASMCs in medium with differentiation supplement (DS), increased mRNA levels of *CSPG4*, *MCAM* and *AOC3* compared to the growth supplement (GS) used in the previous experiments. Clear increases were seen for *AOC3* and *MCAM* (Fig. 5A). *CSPG4* also tended to increase, but this difference did not reach statistical significance (Fig. 5A, P = 0.06).

An important property of MRTFs is that they are controlled by actin dynamics^4,45^. Therefore, we next tested how the *CSPG4*, *MCAM* and *AOC3* transcripts respond to Latrunculin B (LatB), which depolymerizes actin^46^. In initial experiments using either GS or DS media, we did not see an effect of LatB (not shown). We reasoned that this could be due either to the low basal levels of these transcripts in cell culture, or to a preferential drive from MYOCD, which is unresponsive to actin. Consequently, we performed experiments with low-level overexpression of MRTF-A/*MKL1*, which should increase expression levels sufficiently to see LatB-driven reductions. Under these conditions, LatB reduced the mRNA levels of four positive controls (*ACTA2*, *TAGLN*, *SYNPO2*, and *CAV1*, Fig. 5B, left side, 24 h)^12,40,47^. However, *AOC3*, *MCAM* and *CSPG4* levels remained unchanged (Fig. 5B, right side).

It remained possible that *MCAM*, *AOC3*, and *CSPG4* represent comparatively stable transcripts. Therefore, we next tried a cycling protocol over 4 days, in which cells recovered from LatB for 4 h every 24 h to maintain cell viability. In this protocol, *CSPG4*, *MCAM*, and *AOC3* were all reduced by LatB (Fig. 5C, 96 h).

We next examined the Rho-MRTF-SRF inhibitor CCG-1423^48^. Due to the low basal transcript levels, and the slow LatB reductions, we overexpressed MRTF-A/MKL1, and treated cells for 72 h with CCG-1423 (10 μM). Under these conditions, CCG-1423 reduced *AOC3*, *MCAM*, and *CSPG4* levels by > 60% (Fig. 5D). We next constructed time-curves for CCG-1423, which was added at 96 h of MRTF-A/MKL1 transduction (that is when the transcripts should have reached their MRTF-driven peaks). This experiment supported slower mRNA decays for *AOC3*, *MCAM*, and *CSPG4* (red fit, Fig. 5E) relative to established MRTF target transcripts (blue fit, *ACTA2*, *CAV1*, Fig. 5E).

Our inhibitor experiments so far depended on prior overexpression of MRTF-A/MKL1 in vitro. To circumvent this potential caveat, we compared freshly dissected mouse caudal arteries with caudal arteries maintained in organ culture for 96 h in the presence of vehicle or CCG-1423. In organ culture, which should approximate in situ conditions and that does not rely on overexpression of MRTFs, CCG-1423 reduced *Mcam* and *Aoc3* beyond the reduction caused by organ culture alone (Fig. 5F). This was not seen for *Cspg4*, which however declined more sharply with organ culture as such (Fig. 5F). MRTFs are thus likely essential drivers of *Mcam* and *Aoc3* expression in the vascular wall of mice.

MRTFs are antagonized by ternary complex factors (Elk1, Elk3, and Elk4) due to competition for binding to SRF. To examine if this competition applies for *Cspg4*, *Mcam* and *Aoc3*, we next compared wild type (WT) mouse embryonic fibroblasts (MEFs) with MEFs lacking all three Elks (KO). *Cspg4* and *Mcam* were higher in KO MEFs, as predicted, but *Aoc3* was reduced (Fig. 5G). Similarly, overexpression of the atypical ternary complex factor FLI1 in human SMCs reduced *MCAM* and *CSPG4* but increased *AOC3* (Fig. S3). *AOC3* thus deviates from *MCAM* and *CSPG4* with respect to its ternary complex factor regulation.

### MRTFs and cerebrovascular NG2/CSPG4 expression

Recent work examined the transcriptional control of NG2/*Cspg4* in the central nervous system, and it was found that the transcription factors Sox10, Olig2 and Ascl1 activate *Cspg4* expression in glial cells through an enhancer in the first intron (called int1-3b)^44^. This enhancer controlled *Cspg4* expression in Sox10 + cells in vivo but failed to drive expression in pericytes and ECs^44^. Thus, the transcriptional drive on *Cspg4* in pericytes/ECs differs from that in other brain cells. We compared *MYOCD*-*CSPG4* correlations with *SOX10*-*CSPG4*, *OLIG2*-*CSPG4* and *ASCL1*-*CSPG4* correlations in the human nucleus accumbens (GTEx data) and found that *SOX10* (P < 0.0001, R = 0.59), *OLIG2* (P < 0.0001, R = 0.62), *ASCL1* (P < 0.0001, R = 0.63), and *MYOCD* (P < 0.0001, R = 0.35) all correlated with *CSPG4* in the nucleus accumbens. Similar results were obtained in all other brain regions examined (data not shown). Importantly, *SOX10*, *OLIG2*, and *ASCL1* were not represented among transcripts in the extreme of the *CSPG4* correlations in 20 peripheral tissues (see Fig. 1A). These observations, together with previous work^44^, suggest that MRTFs control *CSPG4* expression in pericytes/ECs in the brain, and that *SOX10*, *OLIG2*, and *ASCL1*, while relevant in non-vascular brain cells, play little role in the cerebral vasculature and peripherally. The latter notion was also supported by our finding that *SOX10*, *OLIG2* and *ASCL1* were unresponsive to overexpression of MRTF-A/*MKL1* (c.f. Fig. 4I).

**Figure 6:**
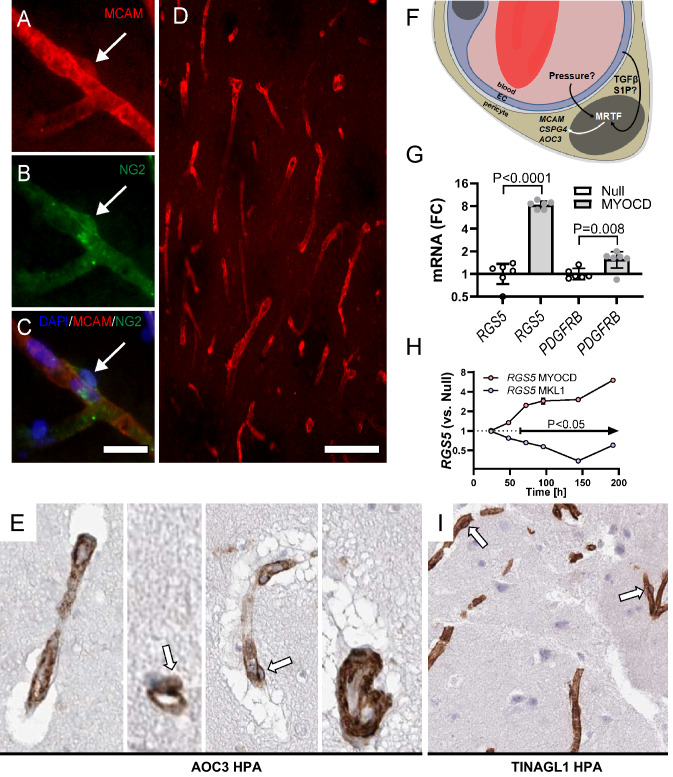
Co-expression of CD146/*MCAM*, NG2/*CSPG4*, and VAP1/*AOC3* in endothelial cells and pericytes in the human brain. Human brain specimen stained with antibodies versus MCAM (**A**) and CSPG4 (**B**) showed co-expression in pericytes and endothelial cells (overlay in **C**). MCAM expression in the human brain was restricted to vascular structures (**D**). Panel (**E**) shows four examples of immunohistochemical staining for AOC3 (brown) in the human brain from the Human Protein Atlas (HPA)^49^. Endothelial cells and pericytes (arrows) in capillaries and larger vessels were positive. Panel (**F**) shows a tentative model for regulation of *MCAM*, *CSPG4* and *AOC3* by MRTFs in pericytes (and endothelial cells) at the human blood–brain barrier. In the illustration, MRTF refers to the three myocardin-related transcription factors MYOCD, MRTF-A/MKL1, and MRTF-B/MKL2. Upstream activators of MRTFs were not examined herein, but some possibilities are given, such as sphingosine-1-phospate (S1P) and transforming growth factor β (TGFβ). Panel (**G**) shows RT-qPCR data for two validated markers of pericytes, *RGS5* and *PDGFRB* (N = 6, 8 days of transduction) in control conditions and after overexpression of MYOCD. Panel (**H**) shows time-course data for the *RGS5* transcript on overexpression of MYOCD and MRTF-A/MKL1, respectively (N = 4 for all times). Null data was generated for each time and used for statistical comparisons but was omitted from the graph for clarity. Panel (**I**) shows staining for TINAGL1 in human cerebral microvessels (from the HPA).

MRTF-driven cerebrovascular transcription in man would be strongly supported if two or more MRTF-regulated proteins are co-expressed in ECs and pericytes. To examine co-expression in cerebral vessels, we stained human brain sections (hippocampus) for NG2/*CSPG4* and CD146/*MCAM*. Both antibodies labelled vascular structures, including ECs and pericytes (Fig. 6A–C). The MCAM antibody did not stain any other cells (Fig. 6D). Due to difficulties with AOC3 detection, we inspected the Human Protein Atlas^49^ for this protein. AOC3 staining of vascular structures was seen in all brain regions examined (Fig. 6E shows four examples). ECs and pericytes (white arrows) were AOC3 positive (Fig. 6E), and SMCs in larger arterioles were also positive (Fig. 6E, right micrograph, bottom). We conclude that co-expression of NG2/*CSPG4*, CD146/*MCAM* and VAP1/*AOC3* in ECs and pericytes support MRTF-driven transcription at the human blood–brain barrier with high probability (estimated P = 0.0005) and distinguishes pericytes and ECs from other NG2 + cells in the human brain.

Our results support a model in which MRTFs exert a transcriptional drive at the human blood brain barrier, regulating the expression of *MCAM*, *CSPG4* and *AOC3* (Fig. 6F). The utility of any model is best determined by its ability to make predictions that can be confirmed. Two predictions were explored. First, we tested whether other commonly used markers of pericytes in the brain are targeted by MRTFs. Indeed, *RGS5* (regulator of G protein signaling 5) and *PDGFRB* (platelet derived growth factor receptor β), which are widely used markers of pericytes, were increased 8 days after MYOCD transduction as shown using RT-qPCR (Fig. 6G). Surprisingly, and unlike the gene trio examined above, MYOCD and MRTF-A/MKL1 regulated *RGS5* in opposite directions (Fig. 6H). The second prediction that we made was that novel markers of ECs and pericytes in the brain can be identified by focusing on MRTF target genes. To gauge this possibility, we used a published RNA-seq dataset for MRTF-A^50^, and examined staining for highly regulated and poorly characterized targets in the Human Protein Atlas. This strategy identified TINAGL1 (tubulointerstitial nephritis antigen like 1), for which staining of cerebral ECs and pericytes was particularly clear (Fig. 6I, in brown). We conclude that two predictions based on our model (Fig. 6F) can be confirmed.

## Discussion

The current work examined regulation of three transcripts that, from a computational point of view, are highly co-expressed across human tissues, forming a closely knit cluster. Correlation analyses implicate MRTF-SRF signaling in regulation of these genes, and we accordingly tested the hypothesis that the gene trio consisting of NG2/*CSPG4*, CD146/*MCAM* and VAP1/*AOC3* is regulated by MRTF-SRF signaling in vitro and in situ. We find that (1) these mRNAs are increased following overexpression of MRTFs, (2) the transcripts are reduced by SRF depletion, and (3) they are affected by ternary complex factors and increased during SMC differentiation. Depolymerization of actin, a well-established control mechanism of MRTFs^4,45^, only slowly reduced *CSPG4*, *MCAM* and *AOC3*, but this was accounted for by slow mRNA degradation compared to other MRTF target genes. Importantly, the MRTF-SRF inhibitor CCG-1423 reduced *Mcam* and *Aoc3* in mouse arteries without prior overexpression of MRTFs, arguing in favor of an essential transcriptional drive from MRTFs on these genes in the intact vascular wall. The human gene loci for *MCAM* and *AOC3* bind SRF or have putative SRF binding sites, and reporter plasmids containing these regions conferred responsiveness to MRTF-A/MKL1. *MCAM* and *AOC3* transcripts responded more strongly to overexpression of MRTFs than the classical target gene *ACTA2*. The large effect-size of MRTFs, along with high quality antibodies (for MCAM in particular), may explain why these gene products have emerged as cell lineage markers. *CSPG4*, *MCAM*, and *AOC3* are expressed on the cell surface, allowing for FACS-based isolation approaches. In recent work, a mouse model for lineage tracing of CD146 + /NG2 + (MCAM + /CSPG4 +) cells was developed^51^. Based on our current findings, this model may tentatively be used to track cells in which MRTFs are particularly active.

A weakness of the present work is that we did not inactivate CArG-boxes responsible for *MCAM* and *AOC3* regulation. However, it seems plausible that one (*AOC3*) or several (*MCAM*) CArGs included in the reporter constructs contribute to regulation. Lack of reporter data with *CSPG4* may be taken to indicate an indirect effect. However, if one entertains the idea of an indirect effect, one will have to reconcile this with our finding that *SRF* is the most highly ranking transcription factor in the human *CSPG4*/*MCAM*/*AOC3* co-expression module, computationally defined using > 1 M correlation analyses. Moreover, an explanation must be provided for the lack of delay in induction compared to the direct target gene *ACTA2*, when a delay by at least 24 h would be expected for an indirect effect. The large effect sizes at the mRNA and protein levels, which clearly match, or even outperform, that of known direct target genes, also need to be accommodated. Therefore, we favor the view that all the genes studied are direct targets of MRTF-SRF-signaling. The smaller effects of MRTF-A on reporter activities compared to the effects at the mRNA levels do however allow for additional activation mechanisms involving for example DNA regions beyond those studied, and/or chromatin remodeling. We found that KDM3A, known to bind MRTFs^42^ and to demethylate the repressive histone mark H3K9Me2^52^, is induced by MRTF-A suggesting chromatin remodeling. Moreover, KDM3A silencing reduced *MCAM* and *AOC3*, consistent with prior work showing involvement of KDM3A in *MCAM* regulation^43^. We therefore propose that the MRTF-SRF complex acts in part by binding to CArGs in the promoters of *MCAM* and *AOC3*, and in part by an indirect mechanism that involves increased KDM3A expression. *CSPG4* stands out by responding less than *MCAM* and *AOC3* to SRF depletion, depolymerization of actin, and KDM3A silencing. Further studies are therefore warranted to unravel the exact mechanisms underlying regulation of *CSPG4* by MRTFs.

The strong correlations between MRTF-SRF and CD146/*MCAM*, VAP1/*AOC3*, and NG2/*CSPG4*, suggest that MRTFs constitute an important, if not major, drive on these genes in many human organs. Our findings moreover support a critical MRTF-SRF drive on these genes in the mouse caudal artery in situ. NG2/*CSPG4* expression in the healthy brain is an exception. In the brain, SoxE and bHLH factors appear to play a dominating role for *CSPG4* expression in NG2 glia^44^. Importantly, our experiments argue against the possibility that SoxE and bHLH factors are indirectly involved in the effects of MRTFs in vascular cells, the reason being that the SoxE and bHLH factors in question were unresponsive to MRTF overexpression. The highly dynamic expression of the lineage markers studied here caution against their use for uncritical enumeration of cerebrovascular cells in e.g. neurodegenerative disease^53^. Indeed, if overall NG2/*CSPG4* levels change with treatment, this could be due to a change in MRTF activity rather than a change in cell numbers. We have relied on NG2 for pericyte counting^54^, but only in combination with simultaneous assessment of cell shape and spatial cell distribution. Given the complex transcriptional influences on these genes, special caution is obviously needed.

Two of the genes studied here (*AOC3* and *MCAM*) behaved as *bona fide* SMC differentiation markers. These markers were increased in an in vitro differentiation paradigm and reduced in organ cultured mouse arteries. A well-established model for SMC differentiation postulates that competition between ternary complex factors (TCFs), such as ELK1, and MRTFs, for binding to SRF is a key event in SMC differentiation^55^. That is, when growth is stimulated and mitogen-activated protein kinases are active, ELK1 binds to the same site on SRF as MRTFs but activates a distinct set of target genes important for growth^55,56^. According to this antagonistic model, the behavior of classical SMC differentiation markers is dictated as much by their MRTF-activation as by their TCF-repression. Our experiments on mouse cells lacking three TCFs showed higher levels of *Cspg4* and *Mcam*, whereas *Aoc3* was reduced*.* Particularly forceful repression by TCFs therefore does not explain why *AOC3* increases more than *MCAM* and *CSPG4* in our in vitro SMC differentiation paradigm.

While previous work has demonstrated an important role of MRTFs at the blood–brain barrier (BBB) in mice, our current findings are the first to demonstrate a role of MRTFs for lineage marker expression at the human BBB. Our findings therefore overcome an important obstacle in clinical translation. Interestingly, in mice, both MRTF-SRF signaling and MCAM control BBB integrity^9,57^. MCAM does this via effects in both ECs and pericytes. In ECs, loss of MCAM reduces Claudin5 expression and disrupts the structure of tight junctions, whereas MCAM loss from pericytes impairs PDGF receptor signaling and pericyte recruitment^57^. Like the loss of MCAM in ECs, inducible deletion of SRF in ECs reduces Claudin5 expression and tight junction integrity^9^. In further support of a function of MRTFs at the human BBB, we show that two widely used markers of brain pericytes, namely *RGS5* and *PDGFRB*, are regulated by MYOCD.

To summarize, the present work establishes NG2/*CSPG4*, CD146/*MCAM* and VAP1/*AOC3* as MRTF-SRF regulated genes. Furthermore, we demonstrate that *MCAM* and *AOC3* activation by MRTF-A depends on promoter regions containing CArG motifs, and on the epigenetic modifier KDM3A. Finally, these genes respond to changes in actin dynamics and ternary complex factors, and to the MRTF-SRF inhibitor CCG-1423.

## Materials and methods

### Correlation analyses using GTEx data

The Genotype-Tissue Expression (GTEx) project is a database open to the scientific community to study relationships between genetic variation and gene expression in human tissues^28^. Summary statistics on the number of tissues for which RNA-Seq data is accessible, and the age, sex and race of donors can be found at https://gtexportal.org/home/tissueSummaryPage. RNA-Seq data was downloaded in Oct 2018 and TMM-normalized as described^47,58^. Pearson analyses, where *CSPG4* was correlated versus all other transcripts across 20 tissues, were made in Excel. Individual correlations of interest were subsequently tested using the Spearman method in GraphPad Prism. The tissues included were tibial artery, aorta, coronary artery, sigmoid colon, the muscular layer of the esophagus and the gastroesophageal junction, tibial nerve, uterus, stomach subcutaneous adipose tissue, terminal ileum, lung, visceral adipose tissue, transverse colon, prostate, breast, skeletal muscle, amygdala, hypothalamus, and ovary. The number of individuals in each group ranged from 100 (amygdala) to 564 (skeletal muscle).

For the analyses described above, we found that *CSPG4*x*MYOCD* correlations in the brain were weaker than elsewhere in the human body. We therefore compared *MYOCD* with three transcription factors previously shown to regulate *CSPG4* (*SOX10*x*CSPG4*, *OLIG2*x*CSPG4*, and *ASCL1*x*CSPG4*)^44^ using six different brain regions. The regions included were cerebral cortex, nucleus accumbens, nucleus caudatus, putamen, hippocampus, and amygdala. *MYOCD*x*CSPG4* correlations were highly significant across the brain regions examined in this analysis (P < 0.0001 throughout).

ChIP-Seq data for SRF (c.f. arrows in Fig. 4A) was examined in the UCSC genome browser (https://genome.ucsc.edu) with the regulation track set to show ENCODE phase 3 binding data for SRF.

### Cell culture, viral transduction and treatments

Human coronary artery smooth muscle cells (hCASMCs, Female 32 years, C-017-5C, Gibco) were purchased from Thermo Fisher and cultured in Medium-231 (M231500, Thermo Fisher) with smooth muscle growth supplement (SMGS, S00725) and antibiotics (50U/ml penicillin and 50 μg/ml streptomycin, Biochrom, A 2212) in a standard cell culture incubator (5% CO_2_). For transfection of promotor reporters, we used HEK293 cells cultured in high glucose DMEM medium with 10% Fetal Bovine Serum (FBS, Biochrom, S0115), and penicillin/streptomycin as above. To stimulate SMC differentiation, we used smooth muscle differentiation supplement (SMDS, S-008-5) instead of SMGS, and cells were maintained for 72 h in either SMGS or SMDS. In first protocol to depolymerize actin (c.f. Fig. 5B) Latrunculin B (Lat B, 100 nM, Calbiochem, 428020) or vehicle (DMSO) were added directly to the medium after 96 h of low-level overexpression of MRTF-A/MKL1 (100 MOI), and cells were harvested after an additional 24 h. In the second protocol (cycles in Fig. 5C), MRTF-A/*MKL1* virus and Latrunculin B/DMSO were added simultaneously in SMGS-free media and maintained for 20 h. The cells were then allowed to recover from LatB and virus for 4 h in SMGS (5%). Thereafter the cycle started over again with LatB/DMSO additions for 20 h and 4 h rests until the cells were harvested at 96 h. In the first CCG-1423 protocol (Fig. 5D), cells were transduced with MRTF-A/MKL1 in 2% SMGS. 10 µM CCG-1423 or the corresponding volume of DMSO was then added at 24 h and cells were harvested at 96 h. A similar design was used for the CCG-1423 time-course studies (Fig. 5E), where cells were harvested at different times with CCG-1423 (10 µM) following initial MRTF-A transduction for 96 h. For trichostatin A treatments, cells were transduced in 2% SMGS with simultaneous addition of 300 nM trichostatin A or the corresponding volume of DMSO, and cells were harvested at 96 h. For transductions, cells were treated with adenoviral vectors encoding MKL1 (MRTF-A, Ad-h-MKL1/eGFP, ADV-215499), MKL2 (MRTF-B, Ad-h-MKL2, ADV-215500), or MYOCD (Ad-h-MYOCD, ADV-216227), all of which were purchased from Vector Biolabs. To silence SRF and KDM3A we used short hairpin constructs (shADV-224323, shADV-212839) and Ad-GFP-U6-shRNA (#1122) as control. Empty adenoviral vector (Ad-CMV-Null, #1300) at the same multiplicity of infection (MOI) served as control in overexpression experiments. Transduced cells were harvested at 96 h unless specified. Mouse embryonic fibroblasts (MEFs) lacking three ternary complex factors (ELK1, ELK3 and ELK4) and the control wild type (WT) cells were a kind gift from Dr. Richard Treisman.

### RT-qPCR

Cells were washed in cold phosphate-buffered saline (PBS, P4417, Sigma-Aldrich) and lysed in Qiasol (Qiagen, #79306). RNA was extracted using the Qiagen miRNeasy mini kit (Qiagen, #217004) and using the QIAcube workstation. Concentration and purity were determined in the Nanodrop 2000c spectrophotometer (Thermo Scientific). RT-qPCR reactions were run using the Quantifast SYBR Green RT-PCR kit (Qiagen, 204156) and primer assays for human (*CSPG4* (QT00120407), *MCAM* (QT00159845), *AOC3* (QT00128716), *KDM3A* (QT00088879), *ACTA2* (QT00088102), *RGS5* (QT00006832), *PDGFRB* (QT00082327), *18S* (QT00199367)), and mouse (QT00159845, QT00120407, QT00128716) transcripts. Primer sequences are considered proprietary information by Qiagen. For amplification we used the StepOnePlus qPCR cycler (Applied Biosystems). 18S was used as reference gene and fold changes were calculated using the Pfaffl method. The relative mRNA level is represented by the official gene symbol in italics in all figures showing RT-qPCR data.

### Protein isolation and western blotting

For protein isolation, cells were washed in ice cold PBS, followed by addition of lysis buffer (80 μl 60 mM Tris–HCl, 2% SDS, 10% glycerol, pH 6.8) and scraping. The protein concentration was determined (BIO-RAD DC protein assay kit, #500-0112), and lysates were adjusted to 1 μg/μl and mercaptoethanol was added (to 5%). Following heating (≈ 95 °C), lysates were stored at – 80 °C. 25 μg of protein was loaded per lane on 4–15% or AnyKd gels (BIO-RAD, #161-0395) along with PrecisionPlus Kaleidoscope markers (BIO-RAD, #161–0395)^59,60^. Gels were run at 200 V until the front ran off using the Tris/Glycine/SDS buffer system (BIO-RAD, #161-0732). For transfer, we used the Trans-Blot Turbo transfer system and 0.2 μm nitrocellulose (BIO-RAD, #170-4159). Following blocking (≥ 2 h in Casein block, BIO-RAD, #161-0782), horizontal membrane strips were cut to allow for detection of multiple targets on the same membranes (uncropped blots for Figs. 1, 2, 3, and 4, in this paper are shown in Fig. S4). Membrane strips were incubated with primary antibody diluted directly in blocking buffer in sealed plastic bags. Bags were tumbled in the cold (4 °C) over night. We used the following primary antibodies CSPG4 (MAB2029, clone 9.2.27, Millipore), MCAM (SAB5600062, Sigma Aldrich), AOC3 (MAB3957, R&D Systems, and SAB2501957, Sigma-Aldrich), KDM3A (12835-1-AP, Proteintech), H3K9Me2 (4658S, Cell Signaling Technology), Histone H3 (4499S, Cell Signaling Technology), phospho-ERK1/2 (Thr202/Tyr204, 9101S, Cell Signaling Technology), total ERK1/2 (9102S, Cell Signaling Technology), HSP90 (#610418, BD Biosciences), GAPDH (MAB374 from Sigma Aldrich). Membranes were washed (3 × 10 min) in Tris-buffered saline (BIO-RAD, 170-6435) with 0.1% Tween (BIO-RAD, 161-0781), incubated with HRP (horse radish peroxidase)-conjugated secondary antibodies (1:10,000, Cell Signaling Technology, #7074S, 7076S) for 2 h, and washed again. For detection we used West Femto substrate (Thermo Fisher Scientific, #34096) and the Odyssey Fc Imager (LI-COR Biosciences). Bands were normalized to GAPDH or HSP90 in the same lane. The mean of the MRTF-A/*MKL1*-transduced samples was set to 100%.

### Confocal imaging

Cultured smooth muscle cells were fixed using 4% PFA for 20 min. 1% BSA, 1% goat serum, and 0.05% saponin in PBS was used for blocking/permeabilization and antibody dilution. Antibodies were used at a dilution of 1:300, and cells were incubated for 1–2 h with each antibody, followed each time by washes in PBS (3 × 5 min). Confocal images were acquired using the Nikon A1 plus instrument and a 60 × Apo DIC oil immersion objective (NA = 1.40, Nikon Instruments Inc.) using appropriate filter sets^36^. Images were acquired in a randomized fashion with Laboratory Imaging (NIS-elements, version 4.50.02). Analysis and quantification was done using FIJI/ImageJ.

### Immunofluorescence staining of human brain tissue

Cerebral MCAM/CSPG4 staining was performed on hippocampal samples from one individual with no cognitive deficits (Netherlands Brain Bank (NBB)). Samples were post fixed in 4% paraformaldehyde for 14 h after autopsy and left in phosphate buffered saline (PBS) containing 30% sucrose for 4 days. Specimens were cut into 30 μm free floating sections. The sections were blocked for 1 h in blocking solution (KPBS with 5% goat serum (Jackson Immunoresearch) and 0.25% triton) and thereafter incubated with antibodies against MCAM (SAB5600062, Sigma Aldrich) and NG2 (cocktail of clone B5, ATCC, kind gift from Dr. William Stallcup, and clone 9.2.27, Millipore) overnight at 4 ºC. The following day the sections were incubated with Dylight 594 goat-anti-rabbit and Alexa 488-goat-anti-mouse (Thermo Fischer Scientific) for 2 h at RT, followed by incubation in Sudan black (1% in 70% ethanol) (Sigma-Aldrich) for 5 min, and mounting with Vectashield Set mounting medium containing DAPI (Vector Laboratories)^61^. Written informed consent for research use of brain tissue and clinical data was obtained from patients or next of kin in accordance with the International Declaration of Helsinki^62^. The medical ethical evaluation committee of VU Medical Centre, Amsterdam, approved methods for brain tissue collection. Approval of the study was obtained from the regional ethical review panel in Lund.

We examined immunohistochemical staining for AOC3 using the human protein atlas^49^. Two antibodies were represented (HPA000980 and CAB025797) showing similar overall staining patterns. Staining for CAB025797 was stronger and crisper and was therefore used for illustrating AOC3 positive pericytes and endothelial cells in different brain regions.

### Promoter reporter assays

A commercial promoter reporter plasmid for *AOC3* was used (HPRM40636 NM_001277731, GeneCopoeia). For *MCAM*, two promotor reporter plasmids containing binding sites for SRF (either S1–S3 or S2–S4, see Fig. 4A) were custom made (S1–S3, chr11:119186074–119189529; S2–S4 chr11:119186882–119191827, GeneCopoeia). Due to transfection difficulties with human SMCs, HEK293 cells were used. One day after seeding, antibiotic-free DMEM media (10% FBS) was added to the cells. The different luciferase reporter plasmids (0.25 μg, GeneCopoeia) were transfected together with p3xFLAG-MKL1 plasmid (0.25 μg, Addgene #11978) using the manufacturer’s protocol for Lipofectamine 2000. 72 h after transfection, the medium was collected and the luciferase activity, as well as the released alkaline phosphatase, were measured using Secrete-Pair Dual Luminescence Assay Kit (GeneCopoeia)^58^.

### Organ cultured mouse caudal arteries and ethics statement

8–12-week-old wild type C57Bl/6 mice of both sexes from ongoing breeding efforts were euthanized with carbon dioxide. The abdominal side of the tail was marked, and the tail was cut and transported to the lab in ice cold HEPES-buffered Krebs solution (135.5 mM NaCl, 5.9 mM KCl, 1.2 mM MgCl_2_, 2.5 mM CaCl_2_, 11.6 mM glucose, 11.6 mM HEPES, pH 7.4). The skin and the underlying fascia on the abdominal side of the tail were cut under a dissection microscope. As much as possible of the tail artery was then removed and cleaned. Each artery from 6 animals was cut in three pieces. One piece was immediately frozen while the remaining two pieces were blindly assigned to organ culture with vehicle (DMSO) or the MRTF-SRF inhibitor CCG-1423 (10 µM). DMSO or CCG-1423 was added to the culture medium before immersion of the arterial segments. The sample size of n = 6 in each of the three groups was based on empirical observations, no values were excluded, and each CCG-1423 treated segment had a vehicle control from the same animal. Following organ culture, tail arteries were frozen, RNA was isolated, and mRNA levels of *Mcam*, *Aoc3*, and *Cspg4* were determined by RT-qPCR. All animal experiments were approved by the Ethical Review Board at Lund University (approval number M4-26) and adhere to guidelines (ARRIVE 2.0) and regulations for animal experimentation.

### Statistics

Statistical tests were made using Log2-transformed expression data. For pairwise comparisons we used student t-test. Multiple comparisons were made using one-way ANOVAs followed by the Bonferroni or Dunnett post-hoc tests. For individual correlation analyses we used the Spearman method. All statistical calculations were made in GraphPad Prism.

## Supplementary Information

Supplementary Information

## Data Availability

The datasets generated and/or analyzed during the current study are available from the corresponding author on reasonable request. RNA-Seq data from the GTEx Portal is available at https://gtexportal.org, and R-scripts for data download are available from the corresponding author.
