# Supplementary material for: NG2/CSPG4, CD146/MCAM and VAP1/AOC3 are regulated by myocardin-related transcription factors in smooth muscle cells
Source: Sci Rep. 2021 Mar 16;11:5955. doi: 10.1038/s41598-021-85335-x (PMC7966398; doi:10.1038/s41598-021-85335-x)
Supplement: Supplementary file 1 — Supplementary Information [file 41598_2021_85335_MOESM1_ESM.pdf]

NG2/*CSPG4*, CD146/*MCAM* and VAP1/*AOC3* are regulated by myocardin-related transcription factors in smooth muscle cells

Catarina Rippe[1]*, Björn Morén[1]*, Li Liu[1], Karin G. Stenkula[1], Johan Mustaniemi[1], Malin Wennström[2], Karl Swärd[1]

*These authors contributed equally. [1]Department of Experimental Medical Science, BMC D12, SE-22184, Lund, Sweden and [2]Department of Clinical Sciences, Malmö, Lund University, SE-221 84 Lund, Sweden. [3]Department of Urology, Qingyuan People's Hospital, The Sixth Affiliated Hospital of Guangzhou Medical University, Qingyuan, China.

Running title: MRTF-controlled vascular markers

Corresponding author: Karl Swärd

Supplementary figure 1A

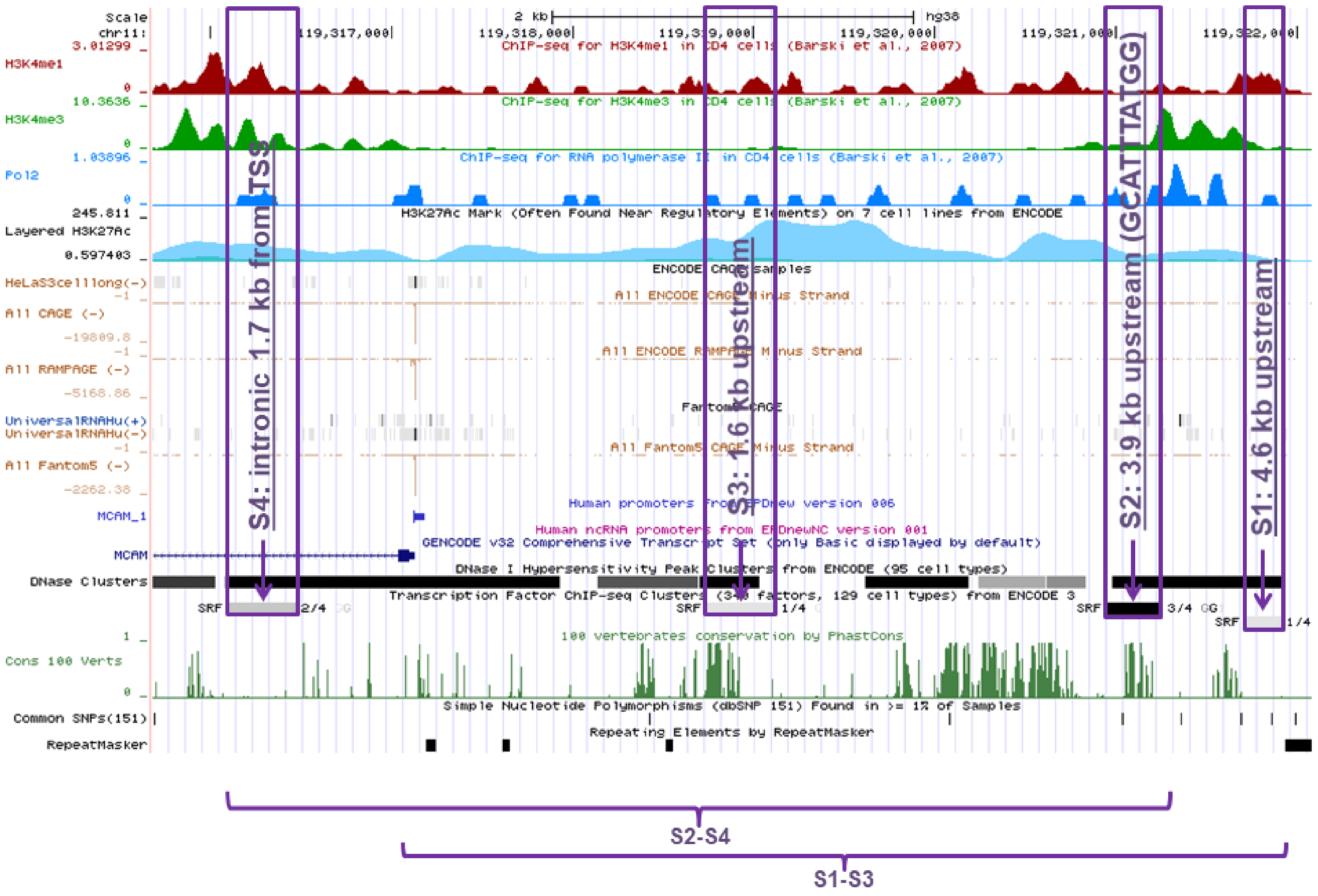

Figure S1A.
SRF binding promotor regions. Chromatin immunoprecipitation-sequencing (ChIP-Seq) data for SRF (ENCODE) was inspected using the UCSC Genome Browser. The figure shows four SRF binding sites (S1, S2, S3, and S4), two of which harbor CArG-boxes (S2 and S4) at the *MCAM* locus. The SRF binding regions are highlighted using purple vertical boxes. DNA regions used for the S1-S3 and S2-S4 luciferase reporters used in the paper are highlighted using purple brackets below the screenshot.

Supplementary figure 1B

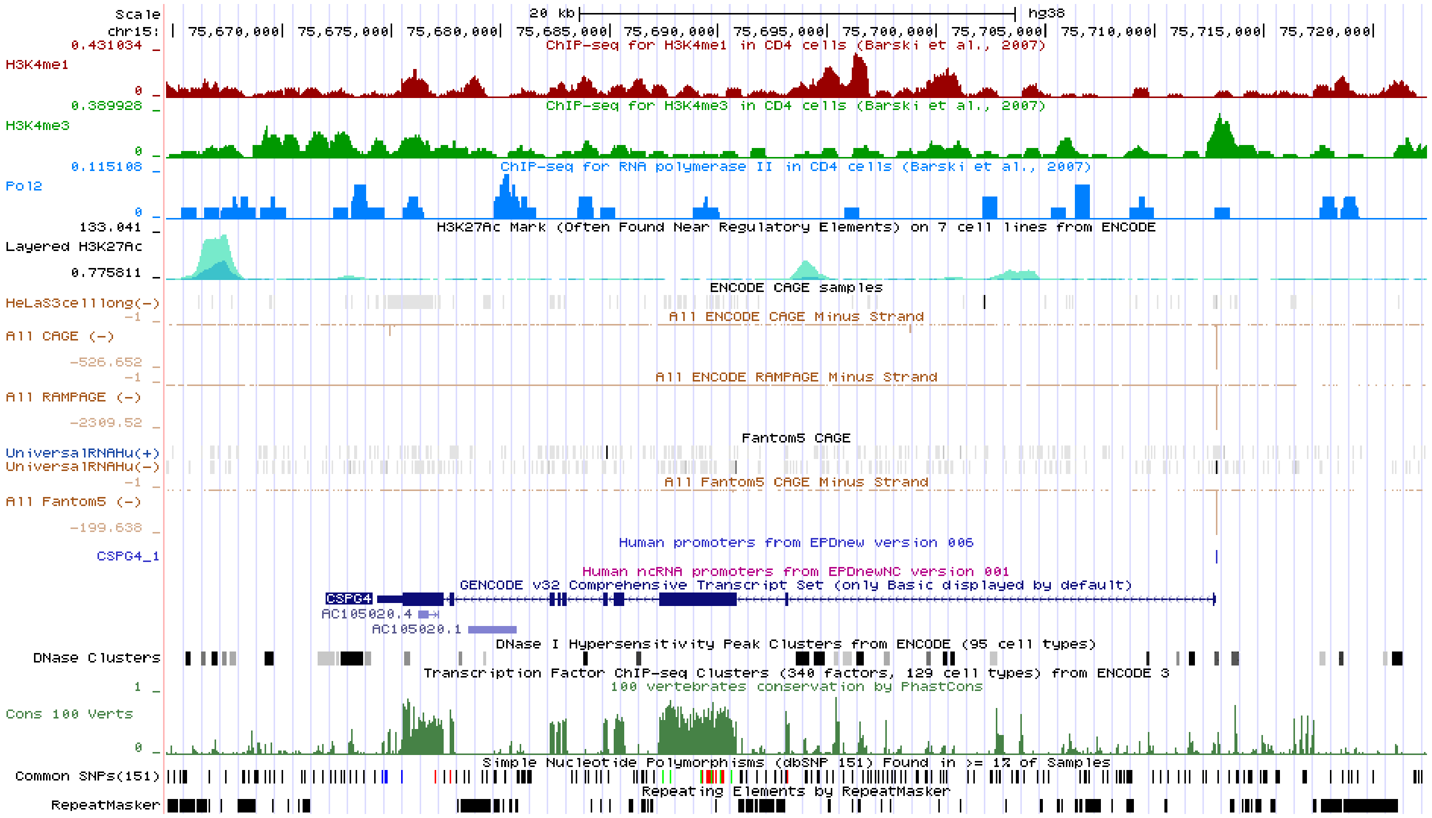

Supplementary figure 1C

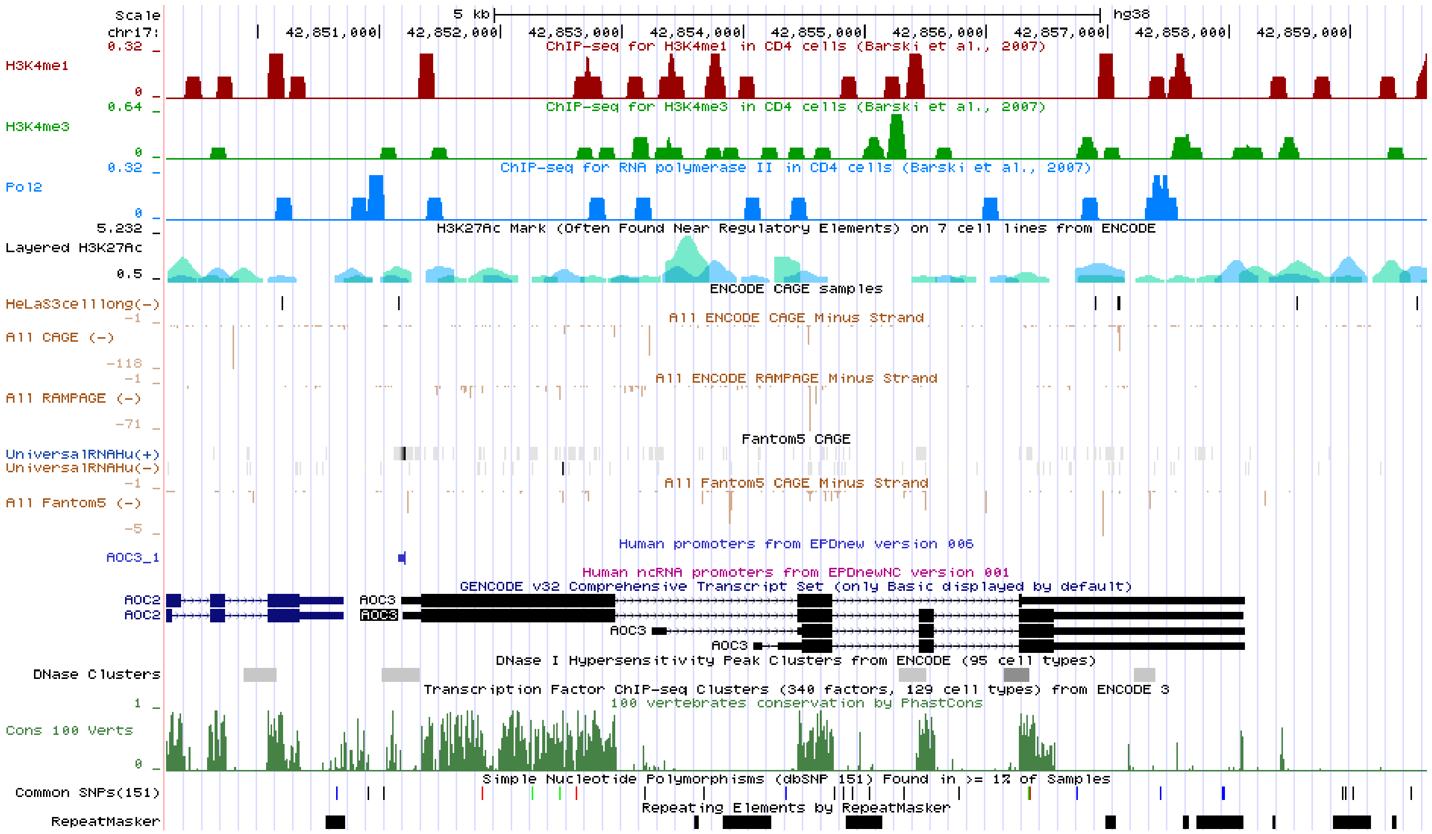

Figure S1B and S1C.
No SRF-binding at the *CSPG4* and *AOC3* loci. SRF binding at the *CSPG4* (B) and *AOC3* (C) loci was inspected using the UCSC Genome Browser. No SRF binding was documented, but the proximal promoter of *AOC3* does contain a computationally defined CArG as described in the text.

Supplementary figure 2

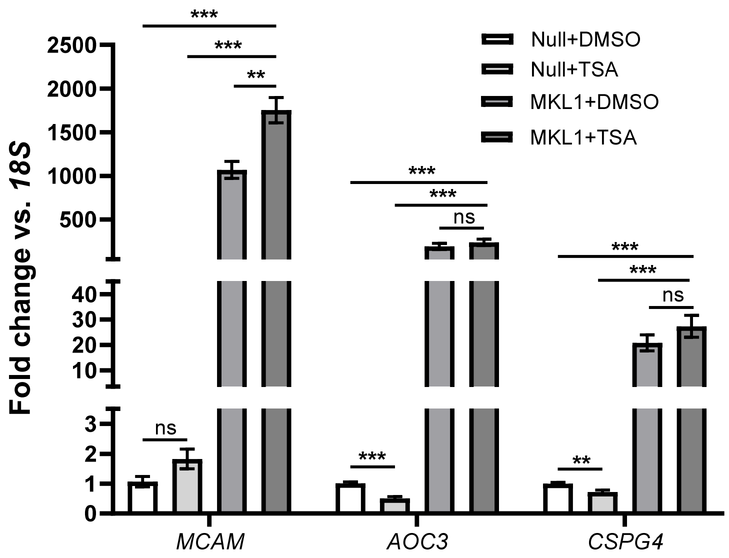

Figure S2.

Trichostatin A, a class I and II histone deacteylase inhibitor, only marginally affects *MCAM*, *AOC3*, and *CSPG4* levels. Treatment of human coronary smooth muscle cells with the deacetylase inhibitor Trichostatin A (TSA 300µM), resulted in a small increase in MRTFA-driven *MCAM* expression, but did not affect *AOC3* or *CSPG4* levels. During basal conditions, TSA marginally reduced *AOC3* and *CSPG4*. mRNA levels were quantified using RT-qPCR. N=6, **P<0.01, and ***P<0.001 for the indicated comparisons.

Supplementary figure 3

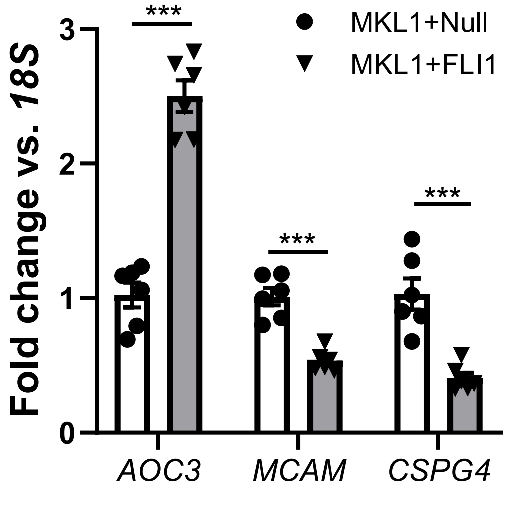

Figure S3.

The ternary complex factor FLI1 increases *AOC* and reduces *MCAM* and *CSPG4* after overexpression of MRTF-A/*MKL1*. Human coronary artery smooth muscle cells were treated with Ad-CMV-MKL1 virus in the presence and absence of Ad-CMV-FLI1 virus. Cells were harvested at 96h. *MCAM*, *AOC3*, and *CSPG4* mRNA levels were quantified using RT-qPCR. Overexpression of *FLI1* reduced *MCAM* and *CSPG4*, but increased *AOC3* expression. *AOC3* thus deviates with respect to its ternary complex factor-dependence. N=6, ***P<0.001 for the indicated comparisons.

Supplementary figure 4 A.

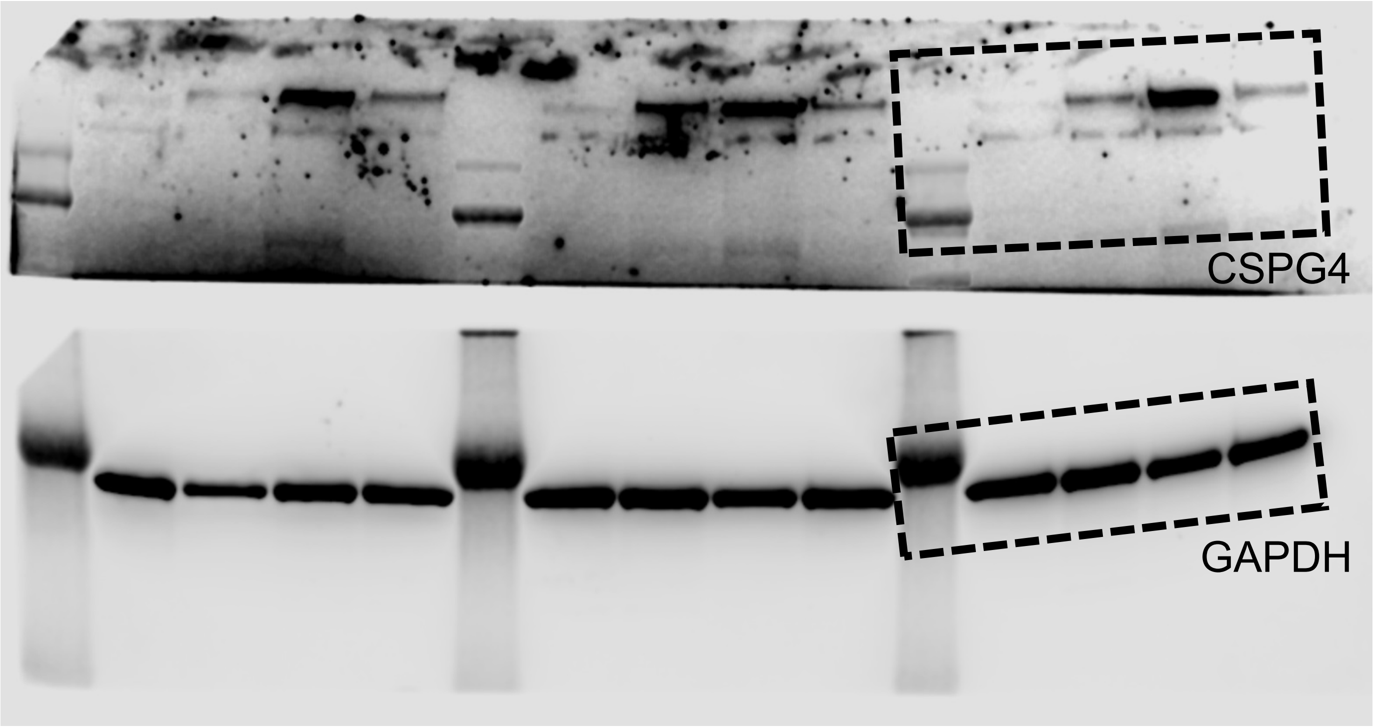

Supplementary figure 4 B.

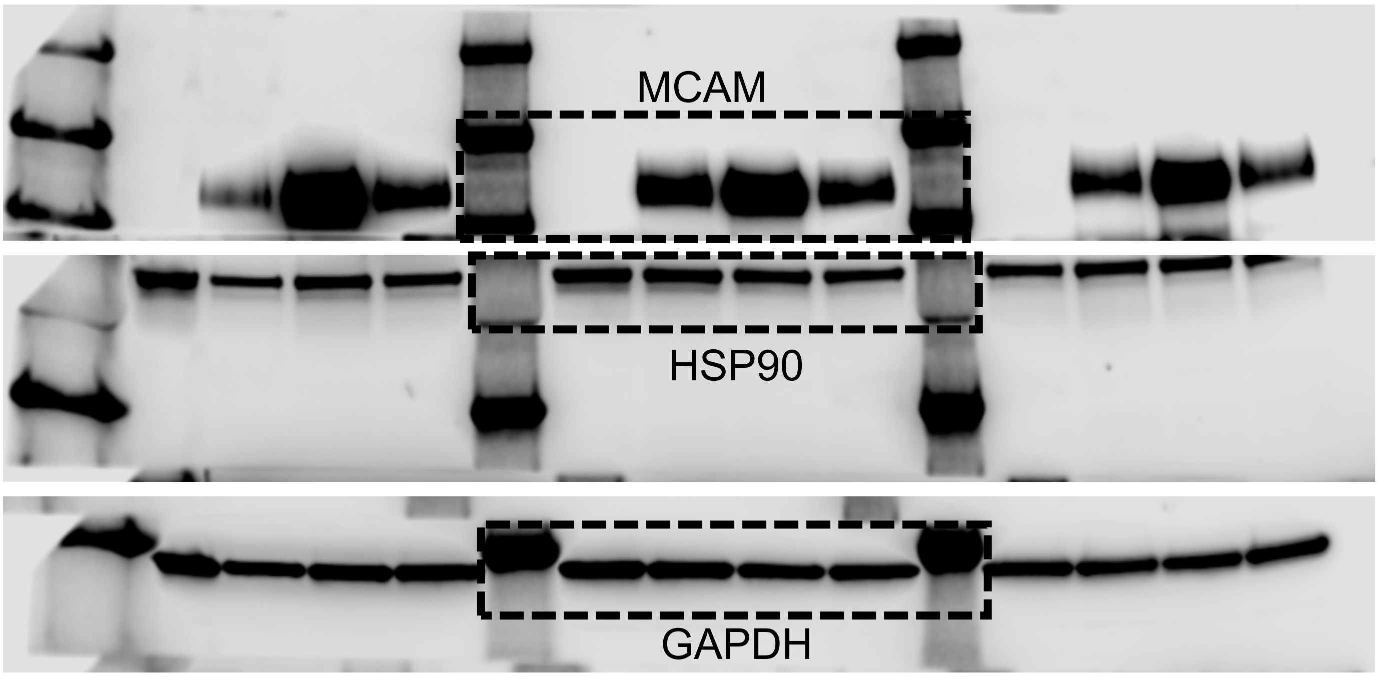

Figure S4A and B

Full length blots for figures 1F (A) and 2F (B) in the manuscript. Crop lines are indicated for the respective targets.

Supplementary figure 4 C.

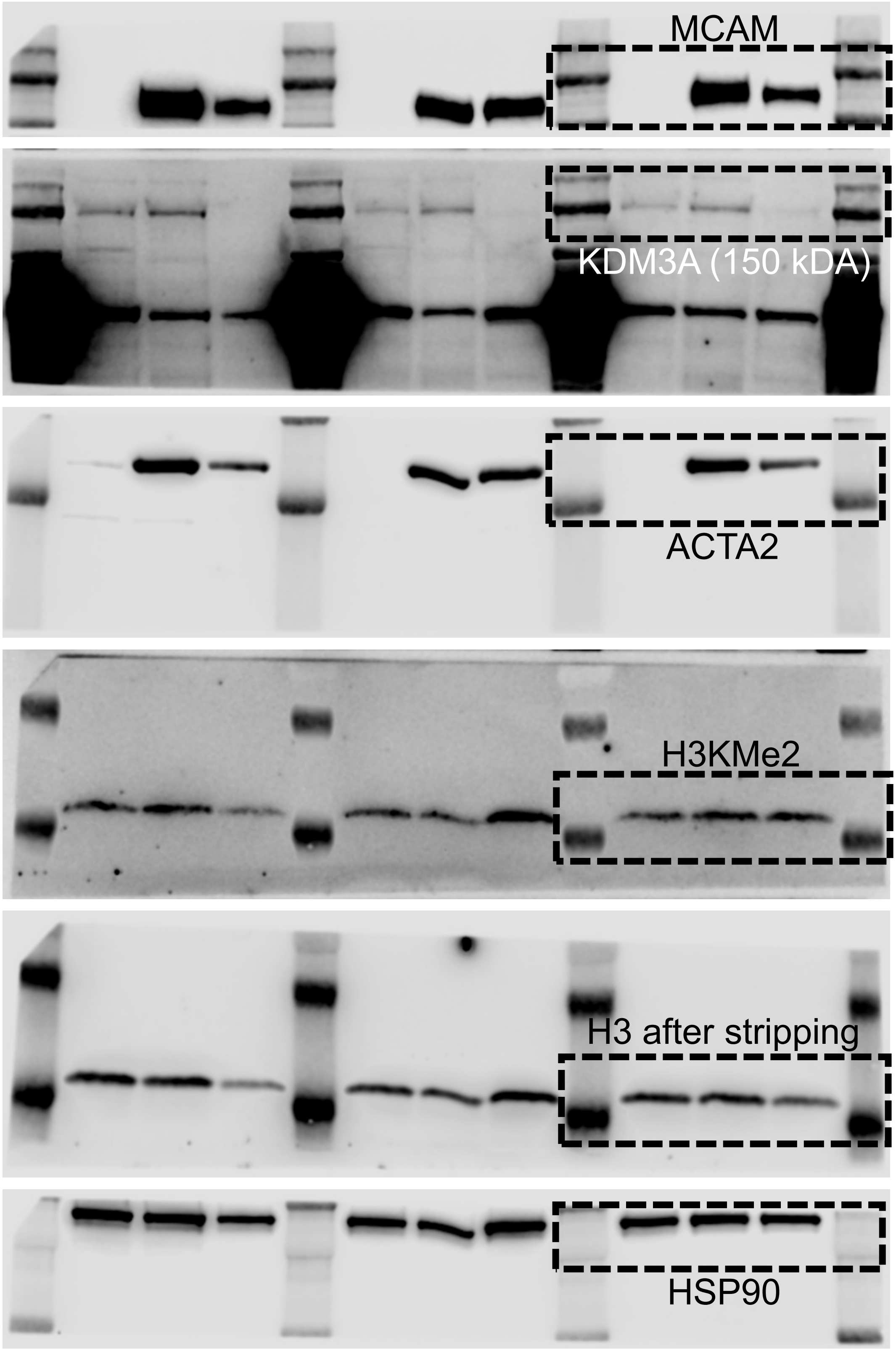

Figure S4C.

Full length blots for figure 4H in the manuscript. Crop lines for the respective targets are indicated.
